# Subacute‐Aggressive‐Onset Chronic Inflammatory Demyelinating Polyradiculoneuropathy as the Initial Presentation in a Pediatric Patient With Systemic Lupus Erythematosus: A 5‐Year Follow‐Up Case Report

**DOI:** 10.1002/pdi3.70028

**Published:** 2025-09-23

**Authors:** Zhiwei Yu, Yuan Xue, Hanyu Luo, Yuhang Li, Siqi Hong, Jiannan Ma, Li Jiang

**Affiliations:** ^1^ Department of Neurology, Children's Hospital of Chongqing Medical University National Clinical Research Center for Child Health and Disorders Ministry of Education Key Laboratory of Child Development and Disorders Chongqing Key Laboratory of Child Neurodevelopment and Cognitive Disorders Chongqing China

## 1 Introduction

Systemic lupus erythematosus (SLE) is a chronic autoimmune disease characterized by the presence of autoantibodies and multisystemic involvement, exhibiting significant clinical heterogeneity. Neuropsychiatric involvement in SLE (NPSLE) predominantly affects the central nervous system. Although peripheral nervous system (PNS) involvement occurs in approximately 7%–20% of adult SLE cases [1, 2], corresponding data in pediatric populations are scarce. Chronic inflammatory demyelinating polyradiculoneuropathy (CIDP) is a rare acquired immune‐mediated inflammatory neuropathy. In line with the European Academy of Neurology/Peripheral Nerve Society consensus guideline revised in 2021, CIDP progresses or relapses for more than 2 months, has electrophysiological or pathological signs of peripheral nerve demyelination, and responds to immunomodulators [3].

The first clinical demonstration of SLE may be CIDP. Meanwhile, the cases reported in children are highly rare [4]. Early recognition of these conditions could help pediatric neurologists and immunologists initiate more suitable immunotherapies, thereby potentially improving the prognosis of affected children. For these purposes, we report a case report of subacute‐aggressive‐onset CIDP as the initial presentation of SLE.

## 2 Case Presentation

A 10‐year‐old girl was admitted (on day 30 after onset) with a month of progressively worsening bilateral lower limb weakness, followed by an upper respiratory tract infection. Initially, the patient experienced difficulty in prolonged walking and navigating stairs. By day 20, this weakness had advanced to a point where she could no longer stand or walk unaided. Three days later, she was only capable of standing with support, concurrently developing numbness in both lower limbs, along with a loss of touch and pain sensation and the ability to sit independently, while retaining normal upper limb movement and touch. Additionally, the patient experienced dizziness, visual impairment, difficulty in urination, and notable personality changes. Continuing to deteriorate until day 35, she exhibited progressive weakness in both upper limbs, accompanied by hoarseness by day 37. At nadir on day 47, she was confined to bed. Therefore, we confirmed the diagnosis of CIDP. On admission, vital signs and general physical examination were normal. The patient's pupils showed an absence of direct and indirect light reflexes, whereas other cranial nerves were not significantly affected. The bilateral Lasegue's test was positive, but signs of meningeal irritation and pathologic reflexes were negative. Muscle strength was graded as 2/5 in the right upper limb, 3/5 in the left upper limb, and 1/5 in both lower extremities, with reduced muscle tone. Knee reflexes were absent bilaterally, with no sensory deficit plane identified. Joint movements of both knees and ankles were restricted, and pain was elicited in the buttocks and posterior aspects of the lower limbs during movement, with heightened pain sensitivity in all four limbs.

Laboratory investigations revealed leukopenia (WBC 4.03 × 10^9^/L, normal 4.3–11.3 × 10^9^/L), decreased PLT (112 × 10^9^/L, normal > 150 × 10^9^/L) and Hb (114 g/L, normal > 115 g/L), normal urea (3.1 mmol/L) and creatinine levels (27 μmol/L), negative CRP (< 8 mg/L) on admission (1 month after onset), and elevated immunoglobulins (IgG 28.7 g/L, IgE 346 IU/mL), normal IgA (2.77 g/L) and IgM (1.98 g/L), as well as significantly reduced complement levels (C3 0.35 g/L, C4 0.05 g/L), which were all measured on day 40 after onset. Urinalysis showed proteinuria (+++) and hematuria (+++), with 25 red blood cells per microliter, whereas kidney function was unremarkable on admission. Cerebrospinal fluid (CSF) analysis on day 32 showed 5 × 10^6^/L nucleated cells, elevated protein (2.35 g/L), and negative cultures. Oligoclonal bands and markers for demyelination (AQP4, MOG, MBP) via cell‐based assay were negative in both CSF and serum, as they were antibodies against gangliosides and cardiolipins and direct and indirect tests for human immunoglobulins. ANCA, myeloperoxidase, proteinase 3, and anti‐glomerular basement membrane antibodies were negative. Antinuclear antibodies showed a nuclear speckled pattern at a titer of 1:1000 and a cytoplasmic speckled pattern at a titer of 1:100, with positive anti‐Sm antibodies and high titers of anti‐nRNP/Sm (U1‐nRNP) and anti‐Ro‐52 antibodies on day 38. Anti‐β2‐glycoprotein was negative. There was no past or current evidence of arterial or venous thrombosis. Nerve conduction studies on day 42 after onset indicated reduced compound muscle action potential (CMAP) amplitudes or absence in multiple nerves of both limbs, with abnormal distal latencies, motor conduction velocities (MCVs), F‐waves, and H‐reflexes, showing a demyelinating pattern (Supporting Information S1: Tables 1 and 2). Magnetic resonance imaging (MRI) of the brain and cervical spinal cord showed no significant abnormalities, whereas thoracic and lumbar imaging revealed enhancement of nerve roots and cauda equina on day 32.

**FIGURE 1:**
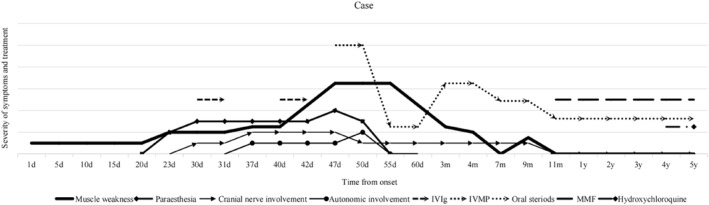
Timeline depicting the clinical presentation, medication regimen, and prognosis of the patient. IVIg, intravenous immunoglobulin; IVMP, intravenous methylprednisolone pulse; MMF, mycophenolate mofetil.

Based on the 2019 EULAR‐ACR criteria [5] for SLE, including lupus nephritis, joint involvement, oral ulcers, decreased C3 and C4 levels, and positive ANA and anti‐Sm antibodies, the diagnosis of SLE was confirmed, supported by central and peripheral nervous system involvement. After considering systemic activity evaluated with SLEDAI‐2K 22 points, the patient's muscle weakness gradually improved with sequential administration of repeated intravenous immunoglobulins (IVIgs 2 g/kg over 2 d) on days 30–31 and days 41–42 and intravenous methylprednisolone pulse (IVMP) on day 47, followed by maintenance therapy with oral steroids. Despite initial emotional instability, she regained the ability to walk and run independently 6 months after onset. Nine months after onset, her condition fluctuated with cessation of steroids, leading to rehospitalization due to recurrent limb weakness and hoarseness. Two months later (11 months after onset), treatment with oral steroids and immunomodulatory therapy with mycophenolate mofetil led to gradual improvement back to normal levels. The patient has been maintained on oral corticosteroids, mycophenolate mofetil, and hydroxychloroquine (initiated 4 years after onset), remaining stable and free of active disease. Currently, the patient continued maintenance therapy with mycophenolate mofetil and hydroxychloroquine and steroids, which have been discontinued for 1 month without any significant flare‐ups or active disease noted at the 5‐year follow‐up (Figure 1).

## 3 Discussion

We reported a highly rare pediatric case presented initially as subacute‐aggressive‐onset CIDP, reaching nadir on day 47; however, on further evaluation, she was found to have juvenile SLE, with a remitting‐relapse course.

The prevalence rate of CIDP in a recent nationwide Japanese survey was assumed to be 3.58 per 100,000 in adults and 0.23 per 100,000 in children, meanwhile highly variable worldwide [6, 7]. In addition, there are no epidemiological data available in China. CIDP in SLE is reported in just 0.2% of patients [4]. In the literature review of pediatric patients, there is one 13‐year‐old boy in Mexico [8], presenting a 7‐month history of CIDP as the first phenotype of SLE and a favorable prognosis after being treated with IVIg, oral steroids, and cyclophosphamide.

The comorbidity mechanisms linking CIDP and SLE involve complex immunological interactions. In CIDP, the primary pathological feature is demyelination driven by macrophage activation. Macrophages, along with T cells, infiltrate the peripheral nerves and nerve roots, contributing to segmental demyelination. This process is initiated, in part, by the deposition of autoantibodies targeting myelin antigens. Complement activation plays a crucial role in this context. The complement system, which bridges innate and adaptive immunity, is activated via the classical, lectin, and alternative pathways. All these pathways converge at C3, leading to the production of effectors such as C3a, C3b, C5a, and ultimately, the membrane attack complex (MAC and C5b‐9). Furthermore, the upregulation of adhesion molecules and proinflammatory cytokines enhances blood–nerve barrier permeability, allowing immune cells and soluble mediators, including antibodies and complement components, to access neural tissues [9]. In SLE, dysregulation of the complement system is a common phenomenon. Although complement fragments such as C3b are essential for clearing cellular debris and preventing autoimmunity, excessive MAC formation can drive tissue damage and further amplify immune responses [10].

Concurrently, SLE is characterized by a breakdown in B‐cell tolerance, leading to the production of a broad spectrum of autoantibodies, including ANA, anti‐Sm, anti‐dsDNA, aPL, and anti‐β2GPI [10]. These autoantibodies can potentially induce neuroinflammatory responses by releasing different neuropeptides such as calcitonin gene‐related peptides, substance P, etc., and axonal or myelin damage through potentially producing anti‐myelin autoantibodies and targeting molecular mimicry of neural structures [11]. Although a small subset of CIDP patients demonstrates antibody responses to myelin proteins (e.g., P0, P2, and PMP‐22) and, more recently, to nodal/paranodal proteins such as NF155, CNTN1, and Caspr1, these antibodies are not consistently detected across all cases. This diverse autoantibody profile underscores the heterogeneity of CIDP pathogenesis [9]. Notably, although clinical studies have not consistently found a correlation between anti‐ganglioside antibodies and peripheral nervous system manifestations in SLE, emerging data suggest a potential role for anti‐Ro antibodies in peripheral neuropathy among patients with SLE and lupus nephritis [1]. Additionally, we found negative evidence of anti‐ganglioside, anti‐β2GPI, anticardiolipin, or ANCA antibodies in our patient. These observations highlight that, despite the identification of several autoantibodies, a definitive pathogenic antibody linking SLE and CIDP has yet to be established, indicating that further research is necessary to unravel these complex immunological mechanisms [11].

Traditionally, glucocorticoids combined with or without IVIg have formed the cornerstone of treatment in patients with SLE‐associated CIDP. Julio et al. [4] conducted a comprehensive review of cases reported between 1974 and 2019 and identified that among the 19 patients of SLE with CIDP, there are two pediatric cases. The 13‐year‐old boy received IVIg, corticosteroids, and cyclophosphamide, whereas the 17‐year‐old girl was managed with corticosteroids, cyclosporine, and plasmapheresis; both cases demonstrated favorable responses. More recently, Wang et al. [12] reported another cohort, which included 9 cases of SLE with Guillain–Barré syndrome (*n* = 6) and CIDP (*n* = 3). When these cases are combined with our current report, a total of 23 patients with SLE‐associated CIDP have been described. Within this pooled cohort, 17 out of 23 patients received IVIg, 19 received corticosteroids, 5 underwent plasmapheresis, and 13 received additional immunosuppressive therapies (with agents including cyclophosphamide in 12 patients, cyclosporine in 2, azathioprine in 4, rituximab in 1, mycophenolate mofetil in 2, and methotrexate in 2). Notably, all but one patient showed significant clinical improvement, underscoring the potential benefit of early and aggressive immunomodulatory treatment.

In our case, the patient initially responded well to a regimen of IVIg, IVMP, and oral corticosteroids. However, during the steroid maintenance phase, the discontinuation resulted in clinical fluctuations and exacerbations. Subsequent administration of mycophenolate mofetil and hydroxychloroquine led to stabilization, further highlighting the need for specified immunosuppressive strategies in cases where SLE activity significantly alters the clinical course compared to isolated CIDP.

In conclusion, we reported an extremely rare pediatric case of SLE initially presenting with CIDP. This case emphasizes the importance of recognizing CIDP as a potential early presentation of juvenile SLE and highlights the need for clinicians to consider immunosuppressive therapies as add‐on options during follow‐up for those differing from the pure CIDP disease progression and treatment. Future investigations are warranted to clarify the immunopathogenic mechanisms linking SLE and CIDP and to develop personalized treatment strategies, ultimately improving outcomes for affected patients across diverse populations.

## Author Contributions

**Zhiwei Yu:** conceptualization, investigation, methodology, project administration, writing – original draft. **Yuan Xue:** data curation, investigation, visualization. **Hanyu Luo:** data curation, investigation, visualization. **Yuhang Li:** data curation, investigation, visualization. **Siqi Hong:** data curation, investigation, visualization. **Jiannan Ma:** conceptualization, project administration, supervision, writing – review and editing. **Li Jiang:** conceptualization, project administration, supervision, writing – review and editing. All authors approved the version to be published.

## Ethics Statement

The ethics committee of Children's Hospital of Chongqing Medical University authorized this study (approval No. 108, 2024). Informed consents for research and publication were obtained from the patient's guardians.

## Conflicts of Interest

The authors declare no conflicts of interest.

## Supporting information


Supporting Information S1

## Data Availability

The clinical data in this research were sourced from medical records. Additional supporting data are available from the corresponding author upon reasonable request.
