# Supplementary material for: Subacute‐Aggressive‐Onset Chronic Inflammatory Demyelinating Polyradiculoneuropathy as the Initial Presentation in a Pediatric Patient With Systemic Lupus Erythematosus: A 5‐Year Follow‐Up Case Report
Source: Pediatr Discov. 2025 Sep 23;3(3):e70028. doi: 10.1002/pdi3.70028 (PMC12483291; doi:10.1002/pdi3.70028)
Supplement: Supplementary file 1 — Supporting Information S1 [file PDI3-3-e70028-s001.docx]

**Nerve conduction studies:**

Table 1 Results of motor nerve conduction studies on admission

| Site | Latency (ms) | Amp (mV) | CV (m/s) | Duration (ms) | Stim (mA) |
| --- | --- | --- | --- | --- | --- |
| Left Median |  |  |  |  |  |
| Wrist-APB | 3.29 | 4.6 | -- | 29.6 | 100 |
| Elbow-Wrist | 7.92 | 2.5 | 41.0 (>3 sd) | 27.2 | 100 |
| Right Median |  |  |  |  |  |
| Wrist-APB | 3.50 (>3 sd) | 6.1 | -- | 27.7 | 100 |
| Elbow-Wrist | 7.38 | 4.4 | 49.0 (>3 sd) | 25.3 | 100 |
| Left ulnar |  |  |  |  |  |
| Wrist-ADM | 2.29 | 7.1 | -- | 31.5 | 92.7 |
| Ab.elbow-Wrist | 8.71 | 2.5 | 26.5 (>3 sd) | 24.7 | 100 |
| Right ulnar |  |  |  |  |  |
| Wrist-ADM | 2.17 | 5.4 | -- | 25.8 | 100 |
| Ab.elbow-Wrist | 8.34 | 2.8 | 29.2 (>3 sd) | 18.0 | 100 |
| Left radial |  |  |  |  |  |
| Elbow-EDC | 4.71 (>3 sd) | 4.8 | -- | 21.2 | 100 |
| Axila-Elbow | 11.8 | 2.3 | 16.2 (>3 sd) | 21.8 | 100 |
| Right radial |  |  |  |  |  |
| Elbow-EDC | 5.00 (>3 sd) | 2.1 |  | 19.0 | 100 |
| Axila-Elbow | 11.5 | 2.1 | 15.4 (>3 sd) | 20.5 | 100 |
| Left peroneal |  |  | -- |  |  |
| Ankle-EDB | 8.21 (>3 sd) | 0.97 |  | -- | 100 |
| Ab.knee-Ankle | 14.2 | 0.51 | 38.4 (>3 sd) | 18.2 | 100 |
| Right peroneal |  |  |  |  |  |
| Ankle-EDB | 7.59 (>3 sd) | 0.95 | -- | 30.9 | 100 |
| Ab.knee-Ankle | 15.00 | 0.70 | 31.0 (>3 sd) | 33.8 | 100 |
| Left tibial |  |  |  |  |  |
| Ankle-Abd hal | -- | -- | -- | -- | 100 |
| Pop Fossa-Ankle | 16.2 | 5.6 | -- | 24.7 | 100 |
| Right tibial |  |  |  |  |  |
| Ankle-Abd hal | -- | -- | -- | -- | 99.8 |
| Pop Fossa-Ankle | 15.8 | 5.8 | -- | 15.5 | 99.8 |

Table 2 Lower limb motor nerve conduction studies at 8-month follow-up

| Site | Latency (ms) | Amp (mV) | CV (m/s) | Duration (ms) | Stim (mA) |
| --- | --- | --- | --- | --- | --- |
| Left peroneal |  |  |  |  |  |
| Ankle-EDB | 6.42 (>3 sd) | 4.2 | -- | 24.6 | 83.6 |
| Ab.knee-Ankle | 13.2 | 4.2 | 38.3 (>3 sd) | 23.4 | 69.2 |
| Right peroneal |  |  |  |  |  |
| Ankle-EDB | 6.29 (>3 sd) | 3.0 | -- | 23.4 | 91.4 |
| Ab.knee-Ankle | 13.0 | 2.7 | 40.2 (>3 sd) | 28.8 | 100 |
| Left tibial |  |  |  |  |  |
| Ankle-Abd hal | 5.21 | 4.6 | -- | 27.8 | 100 |
| Pop Fossa-Ankle | 11.9 | 4.1 | 41.9 (>2 sd) | 32.3 | 99.3 |
| Right tibial |  |  |  |  |  |
| Ankle-Abd hal | 5.59 (>2 sd) | 6.9 | -- | 28.8 | 100 |
| Pop Fossa-Ankle | 13.7 | 5.8 | 35.1 (>3 sd) | 33.7 | 99.4 |
